# Conditional Silencing by CRISPRi Reveals the Role of DNA Gyrase in Formation of Drug-Tolerant Persister Population in *Mycobacterium tuberculosis*

**DOI:** 10.3389/fcimb.2019.00070

**Published:** 2019-03-26

**Authors:** Eira Choudhary, Rishabh Sharma, Yashwant Kumar, Nisheeth Agarwal

**Affiliations:** ^1^Translational Health Science and Technology Institute, NCR Biotech Science Cluster, Faridabad, India; ^2^Symbiosis School of Biological Sciences, Symbiosis International (Deemed University), Pune, India

**Keywords:** *Mycobacterium tuberculosis*, gyrase, DNA damage, SOS, persisters

## Abstract

Drug tolerance in mycobacterial pathogens is a global concern. Fluoroquinolone (FQ) treatment is widely used for induction of persisters in bacteria. Although FQs that target DNA gyrase are currently used as second-line anti-tuberculosis (TB) drugs, little is known about their impact on *Mycobacterium tuberculosis* (Mtb) persister formation. Here we explored the CRISPRi-based genetic repression for better understanding the effect of DNA gyrase depletion on Mtb physiology and response to anti-TB drugs. We find that suppression of DNA gyrase drastically affects intra- and extracellular growth of Mtb. Interestingly, gyrase depletion in Mtb leads to activation of RecA/LexA-mediated SOS response and drug tolerance *via* induction of persister subpopulation. Chemical inhibition of RecA in gyrase-depleted bacteria results in reversion of persister phenotype and better killing by antibiotics. This study provides evidence that inhibition of SOS response can be advantageous in improving the efficacy of anti-TB drugs and shortening the duration of current TB treatment.

## Introduction

Antimicrobial resistance (AMR) is a formidable threat to the mankind. With the emergence of antibiotic-resistant pathogenic strains the burden of mortality due to such infections is manifesting its impact on global economy, which is estimated to cross 100 trillion USD by 2050 (O'Neill, 2014). In context of bacteria, AMR is introduced by either *de novo* mutations or integration of mobile resistance elements in the genome and hence creates a roadblock in the eradication of resistant strains (Durão et al., 2018). Tuberculosis (TB) remains the leading cause of death by single microbial infection which annually kills ~1.3 million HIV negative individuals across the globe. This burden is further escalated by infection with drug resistant strains of the causative pathogen *Mycobacterium tuberculosis* (Mtb), resulting in an incidence of nearly half a million cases due to multi-drug resistant (MDR) strains, of which ~6% are also extensively-drug resistant (XDR) strains (WHO., 2017).

The anti-TB chemotherapy comprises of two phases, the initial intensive phase of 2 months with daily intake of four drugs: rifampicin (Rif), isoniazid (INH), ethambutol (Emb) and pyrazinamide (PZA), and continuation phase with Rif and INH for 4–7 months (Joshi, 2011). While the initial phase is aimed to avoid the emergence of the drug-resistant organisms and to quickly reduce the bacillary load, the subsequent treatment in the continuation phase targets the recalcitrant bacterial subpopulation known as “persisters” that exhibit antibiotic insensitivity without altering their genetic makeup (Hobby and Lenert, 1957; Stewart et al., 2003; Gomez and McKinney, 2004; Jain et al., 2008). The incidence of drug resistant TB is generally attributed to the non-compliance of the regimen; however, survival fitness achieved by the pathogen during persistent state might also contribute in the emergence of AMR (Cohen et al., 2013). Despite the ongoing efforts to combat resistant strains by revitalization of current drug therapy (Sharma et al., 2017), the incidence of resistance against newly discovered anti-TB drugs *viz*. delamanid and bedaquilline has prompted a reassessment of the combinatorial drug therapy followed so far (Hoffmann et al., 2016).

Although multiple model systems have been employed to elicit the formation of persisters such as exposure to antibiotic (Hu et al., 2000; Dhar and McKinney, 2010; Keren et al., 2011) and stress agents (Betts et al., 2002; Deb et al., 2009), the underlying mechanism(s) of the induction of these subpopulation in Mtb is poorly characterized. Treatment with DNA gyrase inhibitor fluoroquinolone (FQ), such as ciprofloxacin, is one of the most common strategies to generate persisters in other bacteria (Hansen et al., 2008; Dörr et al., 2009; Wu et al., 2012; Grassi et al., 2017). Even though FQs are clinically used as second-line anti-TB drugs, their impact on formation of persisters in Mtb is not known.

In order to understand the direct association of DNA gyrase with the induction of persister phenotype in Mtb at the molecular level, herein, we use the CRISPR interference (CRISPRi) approach (Choudhary et al., 2015) to examine the effect of DNA gyrase depletion on physiology of Mtb H_37_Ra (hereafter referred as Mtb). We show that silencing the DNA gyrase expression severely attenuates bacterial proliferation, both *in vitro* and intracellularly. Interestingly, we observe that ~3.5% of total Mtb transcripts are induced upon suppression of DNA gyrase, majority of which are associated with DNA damage stress response including RecA-LexA regulons (Durbach et al., 1997; Brooks et al., 2001; Davis et al., 2002; Boshoff et al., 2003). Despite showing the extreme effect on growth, downregulation of Mtb DNA gyrase significantly stimulates phenotypic tolerance to different first-line anti-TB drugs. Furthermore, chemical inhibition of RecA reveals the involvement of RecA-LexA pathway in the evolution of DNA gyrase-controlled persisters in Mtb. Finally, we show that exposure to RecA inhibitor reverses the effects of DNA gyrase inhibition in Mtb and improves the bacterial killing by anti-TB chemotherapeutic agents.

## Materials and Methods

### Bacterial Culturing

Mtb cells were cultured in Middlebrook 7H9 broth or 7H11 agar supplemented with 1XOADS (Oleic acid [0.054 gm/l], Bovine Serum Albumin Fraction V [5 gm/l], Dextrose [2 gm/l], Sodium Chloride [0.81 gm/l]), 0.02% Tyloxapol, and 0.5% Glycerol. Cultures were grown at 37°C without (on 7H11 agar) or with (in 7H9 broth) shaking at 200 rpm. Wherever required following concentrations of antibiotics were used: 25 μg/ml kanamycin (Kan) and 50 μg/ml hygromycin (Hyg).

### Construction of Knockdown Strains

To achieve the repression of genes *MRA_0005/MRA_0006* (*gyrB/gyrA*), *MRA_2165* (*ftsZ*), and *MRA_2487* (*clpP2*), a pair of complementary oligonucleotides specific to the target ORF near 5′-end (Table S1) were synthesized, annealed and cloned in pGrna, as previously described (Choudhary et al., 2015), and illustrated in Figure S1. The recombinant pGrna plasmid containing gene-specific sgRNAs was transformed into dCas9-expressing Mtb H_37_Ra to generate knockdown strains, namely, *gyr(-), ftsZ(-)*, and *clpP2(-)*, respectively. Suppression was achieved by treatment of bacterial cultures with 20 ng/ml anhydrotetracycline (ATc) for 4 days (unless indicated otherwise).

### Microscopy

#### Confocal Laser Scanning Microscopy

Bacterial cultures containing ~10^8^ cells were pelleted and washed twice with 1X PBS, followed by suspension in 1X PBS and repeated passing through 26-guage needle. For nucleoid staining, bacterial cells were incubated with 5 μg/ml DAPI for 15 min on ice. After incubation, cells were washed with 1X PBS to remove the excess of dye and a thin smear of stained culture was prepared on a glass slide. For staining of neutral lipid (to detect lipid bodies), single cell suspension of bacteria was prepared as described above, followed by incubation with 9 μM Nile red (9-diethylamino-5-benzo[*a*]phenoxazinone) dye for 15 min at room temperature in dark. After staining, bacteria were pelleted, washed with decolorizing solution (0.5% HCl, 70% isopropanol, and 30% water) and 1 X PBS. Single cell suspension of stained bacteria was used to prepare thin smear on glass slide and mounted using anti-fading mounting medium. Images were acquired on Olympus FluoView FV1000 confocal microscope equipped with a 100X (numerical aperture,1.4) oil differentiated interference objective. DAPI and Nile red stained bacteria were viewed at lasers corresponding to 405 and 559 nm wavelengths, respectively. Similar capture time and adjustments were used for all the images. Cells exhibiting more than two foci in Nile red staining were considered lipid bodies (LB)-positive. For measurement of bacterial cell length, differential interference contrast (DIC) images were captured on Olympus FluoView FV1000 microscope and analyzed using FluoView FV1000 software. Each analysis was performed with images from a minimum of 20 different fields comprising of ~300 cells.

#### Scanning Electron Microscopy (SEM)

For SEM analysis, bacterial cultures of control and *gyr(-)* were pelleted and washed twice with 0.1 M phosphate buffer (pH 7.4), followed by single cell suspension of pellets in fixative buffer (2% paraformaldehyde and 2.5% glutraldehyde in 0.1 M phosphate buffer). After overnight incubation at 4°C, cells were washed twice with 0.1 M phosphate buffer and observed under Zeiss EVO40 microscope at the Advanced Instrumentation Research Facility in the Jawaharlal Nehru University, India (https://www.jnu.ac.in/airf).

### Antibiotic Susceptibility Assay

Antibiotic treatment was performed with broth cultures of control and respective knockdown strains after 4 days of treatment with 5 ng/ml ATc at equal OD_600_ of 0.05 to achieve ~50% suppression of gyrase and maintain sufficient growth of bacteria. CFU was estimated in antibiotic-treated or untreated samples at indicated time points by spreading serial dilutions of cultures on 7H11 agar containing Kan and Hyg, after 4 weeks of incubation at 37°C. Percentage viability in drug-treated cultures compared to untreated was calculated to analyze the effect of gene knockdown on susceptibility to a particular antibiotic. Antibiotic susceptibility was also tested by spotting two-fold serial dilutions of bacterial cultures on 7H11 agar plates, followed by 4 weeks of incubation at 37°C. To determine the effect of suramin on susceptibility to antibiotic, initial ATc treatment of control and *gyr(-)* was performed in the presence of 50 μM suramin for 4 days in order to limit the RecA activity prior to drug treatment, followed by adjustment of culture OD_600_ to ~0.05 and subsequent incubation with respective antibiotics and 50 μM suramin for different time points. Cell viability was determined as described above.

### MIC Determination

The MIC_99_ (MIC to inhibit 99% of cell viability) of different drugs against the passaged cultures of control and knockdown strains was determined by micro-titer broth dilution method (Hall et al., 2011). Briefly, the OD_600_ of cultures were adjusted to ~0.05 and incubated with equal volume of different dilutions of antibiotics in 96-well plate (final volume of 100 μl per well). Plates were incubated at 37°C for ~2 weeks. Growth as a visible bacterial pellet at the bottom of well was noted and compared with the growth of untreated control wells. The lowest concentration of antibiotic which prevented pellet formation was considered as MIC_99_.

### Intracellular ATP Measurement

Bacterial cultures were pelleted down, washed twice with 1 X PBS and whole cell lysate was prepared in 1 X PBS by bead beating. Level of ATP was measured in the lysate using Bac Titer-GloTM assay kit as per the manufacturer's recommendations (Promega). ATP concentration in each sample was denoted as relative luminescence units (RLU) normalized to total protein concentration in the lysates.

### RNA Extraction and Microarray Analysis

Total RNA was extracted using RNAiso plus reagent (Takara Bio Inc.) according to the manufacturer's instructions from two independent sets of control and *gyr(-)* strains of Mtb after 4 days of depletion. Fluorescent dye-labeled cDNA were prepared by using 4 μg RNA from both the strains with the help of SuperScript® Direct cDNA Labeling System (Thermo Fisher). For each hybridization, the cDNA probes were labeled with Alexa fluor 555 or Alexa fluor 647 (Life Technologies) and used in pairs. Complementary DNAs from the first set of samples were prepared using Alexa fluor 555-tagged dCTP for control and Alexa fluor 647-tagged dCTP for the knockdown, whereas for the second set dyes were switched such that control cDNA samples were labeled with Alexa fluor 647 and those from *gyr(-)* were prepared using Alexa fluor 555-tagged dCTP. Mtb-specific microarray slides were commercially procured (Microarray Inc.) that comprise of oligonucleotides specific to 3924 genes representing complete genome of Mtb in triplicates. Scanning of slides and data analyses were performed as described previously (Thakur et al., 2016). Mean fluorescence intensities of each of the 3924 spots from three different hybridizations per array were analyzed after normalization with total fluorescence intensities. Fold-change in expression levels was subsequently determined using the normalized fluorescence intensities of green and red channels. Average of values from two biological repeat experiments were used to identify genes which exhibit >two-fold difference in expression between the two strains with *P*-values of 0.05 or less, as determined by paired Student's *t*-test and false discovery rate (FDR) of <5%, as calculated by Benjamini–Hochberg procedure.

### Quantitative Real Time Reverse Transcription PCR

First strand cDNA synthesis was performed with 1 μg total RNA after DNase I treatment using random hexamer primers and Superscript III reverse transcriptase (Invitrogen Life Technologies), according to the manufacturer's recommendations. PCR was performed with 50 ng cDNAs and SYBR Green PCR Master Mix (Applied Biosystems) using gene-specific primer pairs (Table S1), and real-time quantification was carried out using the ABI 7500 Fast Real-Time PCR System (Applied Biosystems) as instructed by the manufacturer. Expression level of different genes in the DNA gyrase depleted strain was estimated relative to their expression in ATc-untreated control after normalizing with the change in expression level of a housekeeping gene *sigA*, as described previously (Thakur et al., 2016).

### Fluorescence-Activated Cell Sorting (FACS) Analysis

For nucleoid morphology analysis by FACS, DAPI-stained cells equivalent to ~10^5^ CFU/ml in 1 X PBS were passed through 26 gauge syringe needle and acquired on BD FACSCanto II (BD Biosciences) at low aspiration speed. DAPI was excited with violet laser and detected with 450/50 nm bandpass emission filter. Total of 10,000 events were acquired and gating was set using unstained cultures of control and *gyr(-)* by forward scatter (FSC) and side scatter (SSC) of light. Subsequently, bacterial cells were positioned on FSC-A and SSC-A scatter plot. Data acquisition was performed by BD FACSDIVA™ software (BD Biosciences) and analyzed using FlowJo v10.0.6 software (FlowJo, LLC). Distinctive DAPI staining was evaluated based on median fluorescence intensity obtained from control and *gyr(-)* strains.

### Macrophage Infection

For macrophage infection, THP1 cells were seeded in 24-well plates at a density of 2 × 10^5^ cells per well and differentiated using 50 nM phorbol myristate acetate (PMA). After 24 h of differentiation, PMA was removed and cells were infected with control and *gyr(-)* at 1:2 MOI (macrophage: bacteria) in RPMI medium containing 10% heat-inactivated fetal bovine serum (FBS). After 4 h of incubation, cells were washed with pre-warm 1 X PBS and replenished with RPMI-FBS containing amikacin (50 μg/ml) for 1 h to kill extracellular bacteria. Subsequently cells were washed and maintained in RPMI-FBS medium containing 200 ng/ml ATc throughout the experiment. At different time points cells from two wells were harvested by using 1 X PBS+0.1% Triton X100 and incubated on ice for 5 min in order to release intracellular bacteria. Cell debris were removed by centrifugation and bacterial supernatant was serially diluted in 1 X PBS and spread on 7H11 agar plates for CFU enumeration after 4 weeks of incubation at 37°C.

### Analysis of Metabolites

#### Metabolite Extraction

Metabolites were extracted from ATc-treated empty vector control and gyrase knockdown strains using ~2.0 OD_600_ equivalent culture pellets suspended in 1 mL of ice-cold 80% methanol (in water) by multiple freeze thaw approaches (Sellick et al., 2011). Extracted metabolites were vacuum dried and stored at −80°C till further use.

#### Estimation of Metabolites

Samples were reconstituted in 15% methanol before analysis by liquid chromatography mass spectrometry (LC-MS). All data were acquired on the Orbitrap Fusion mass spectrometer (Thermo Scientific) equipped with heated electrospray ionization (HESI) source. Data were acquired on positive and negative modes using the spray voltage of 4,000 and 35,000 volt, respectively, at 120,000 resolution in MS mode and 30,000 resolution in data-dependent MS2 scan mode. Sheath gas and auxiliary gas were set to 42 and 11, respectively. Mass scan range of 50–1,000 m/z, ACG target at 200,000 ions and maximum injection time of 80 ms were used for MS, whereas ACG target was set to 20,000 ions and maximum injection time of 80 ms was used for MS/MS. Cell extracts were separated on UPLC using HSS T3 column (100 × 2.1 mm i.d, 1.7 μm, Waters) maintained at 40°C. The mobile phase A was 0.1% formic acid in water and mobile phase B was 0.1% formic acid in acetonitrile. The elution gradient was used as follows: 0 min: 1%B+99%A; 1 min: 15%B+85%A; 4 min: 35%B+65%A; 7–9 min: 95%B+5%A; and 10–14 min: 1%B+99%A. The sample injection volume was 5 μl and the flow rate of 0.3 ml/min was maintained throughout the sample elution.

#### Data Processing and Identification of Metabolites

All acquired data were processed using compound discoverer 2.1 software (Thermo Scientific) with the default settings to carry out metabolite identification and quantitation. The untargeted metabolomics workflow of compound discoverer 2.1 was used to perform retention time alignment, feature detection, and elemental composition prediction. Experimental blank runs acquired during data acquisition were used to remove background features arising from mobile phase. Identification of metabolites was performed primarily on the basis of in-house library of compounds for accurate mass, fragmentation, and retention time match, which were also verified on the basis of the accurate mass and fragmentation information available on mzCloud database with compound discoverer 2.1 (Thermo Fisher Scientific).

## Results

### Mtb Growth Is Drastically Affected by Suppression of *gyrB-gyrA* Operon

**Figure 1:**
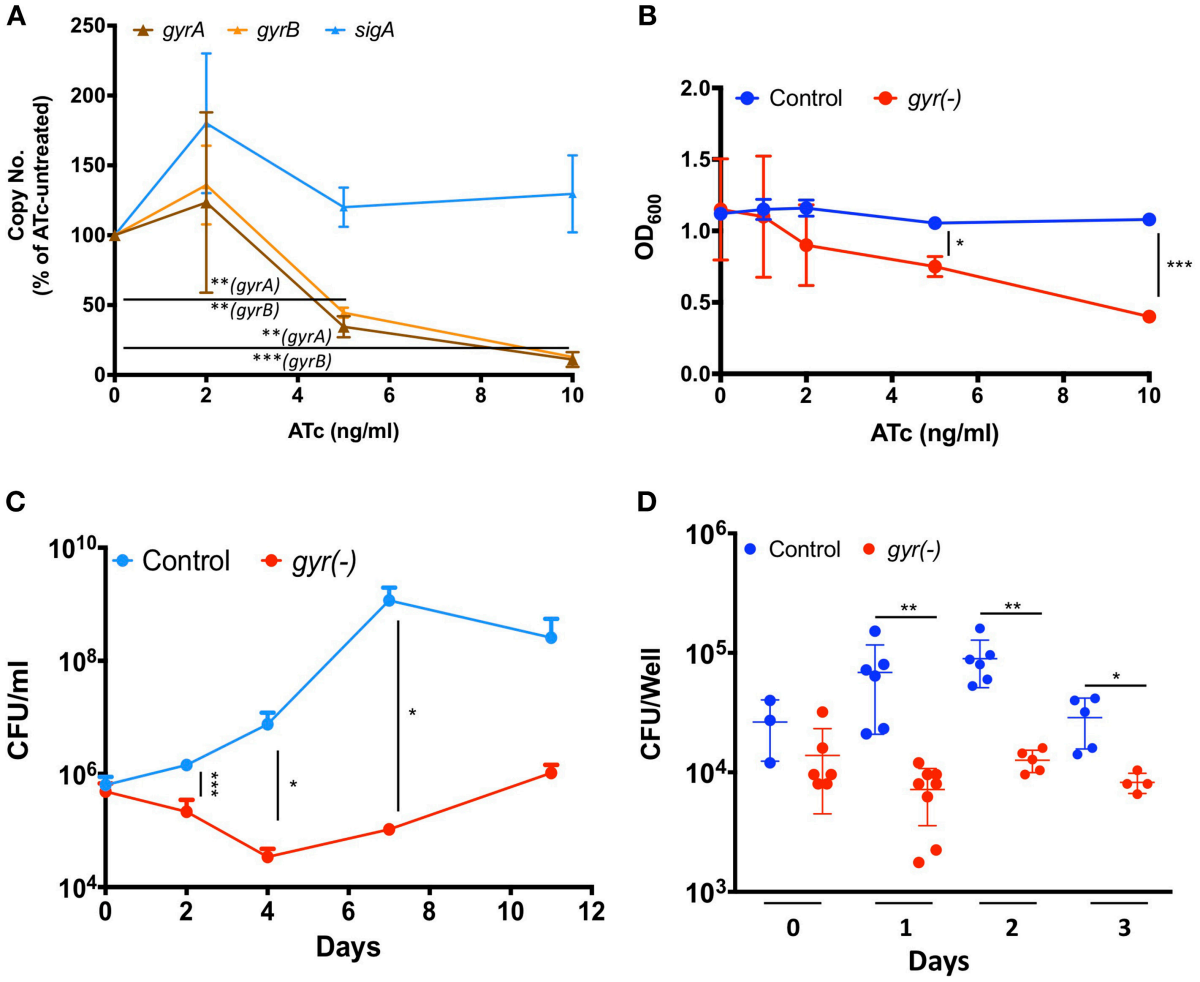
DNA gyrase is indispensable for growth of Mtb H_37_Ra. **(A)** Effect of CRISPRi-mediated silencing on expression of DNA gyrase-encoding genes. Transcript levels of *gyrA* and *gyrB* were measured in *gyr(-)* strain by qRT-PCR following treatment with increasing concentrations of ATc for 4 days. Shown is percentage modulation in copy number of respective transcripts in comparison to ATc-untreated control. Error bars represent the SD from four measurements. **(B)** Influence of dose-dependent suppression of DNA gyrase-encoding genes on growth of Mtb. *in vitro* growth of empty vector containing control and *gyr(-)* was determined by measuring OD_600_ of bacterial cultures treated with different concentrations of ATc for 4 days. Error bars represent the SD from four measurements. **(C)** Effect of DNA gyrase suppression on time-dependent growth kinetics of Mtb. The *in vitro* growth of empty vector containing control and *gyr(-)* was determined at designated time points by CFU enumeration of viable bacteria in cultures treated with 50 ng/ml ATc. Error bars represent the SD from at least three measurements. **(D)** Comparative analysis of intracellular growth profile of control and *gyr(-)* strains of Mtb. Infection of human monocyte-derived macrophages was performed with 1:2 MOI (macrophages: bacteria) for 4 h followed by treatment with 50 μg/ml amikacin to kill extracellular bacteria. Intracellular bacterial load was estimated by spreading serial dilution of macrophage lysates at indicated time points on 7H11-OADS agar plates. Error bars represent the SD from at least three measurements. Statistical significance is determined by paired Student's *t*-test: ^*^P < 0.05, ^**^P < 0.01, ^***^P < 0.001.

DNA gyrase is a heterodimer protein complex comprising of two subunits GyrB and GyrA that are encoded by *gyrB* (*MRA_0005*) and *gyrA* (*MRA_0006*) genes which are clustered in an operon-like fashion in the genome of Mtb. Suppression of DNA gyrase encoding genes in Mtb was achieved by employing the CRISPRi approach (Choudhary et al., 2015; Figure S1), as described in the materials and methods. The resulting strain was annotated as *gyr(-)*. Copy number of both the *gyrB* and *gyrA* transcripts was examined in *gyr(-)* after 4 days of treatment with different doses of anhydrotetracycline (ATc) by quantitative real time reverse transcription PCR (qRT-PCR). Figure 1A shows a gradual reduction in the expression of both the genes with increasing concentration of ATc. While there is 55 and 65% reduction in *gyrB* and *gyrA* levels, respectively, after exposure to 5 ng/ml ATc, further increase in ATc concentration to 10 ng/ml leads to ~90% reduction in expression levels of these genes. Moreover, there is no effect of ATc on the transcript levels of an unrelated control gene *sigA* (Figure 1A). We also notice a similar effect of ATc on *in vitro* growth of *gyr(-)* strain of Mtb which exhibits ~50% reduction in OD_600_ after 4 days of incubation with 10 ng/ml ATc, in comparison to ATc-untreated culture (Figure 1B). Noteworthy to mention that these effects are attributed to only *gyr(-)* strain, as the control strain of Mtb harboring empty plasmid does not exhibit any change in growth profile when cultured in the presence of ATc (Figure 1B).

Next, we examined the growth profile of *gyr(-)* and control strains over a period of two weeks, when inoculated in the 7H9 broth medium containing ATc. Since ATc gets metabolized, here we used a relatively higher concentration (50 ng/ml) of it for constant suppression of *gyrA-B* genes. While the control strain exhibits normal growth, replication of *gyrA-B* depleted Mtb is severely affected by ATc treatment. Quantitative estimation of *gyr(-)* by enumeration of colony forming unit (CFU) reveals 85% reduction in growth on day 2, and ~99% reduction on days 4, 7, and 11 post-ATc treatment when compared with growth of the control bacteria (Figure 1C). These results thus demonstrate that constant suppression of DNA gyrase in Mtb prohibits bacterial proliferation *in vitro*.

Further, we also evaluated the effect of DNA gyrase suppression on intracellular survival of Mtb in THP-1 derived macrophages. Infection was performed with control and *gyr(-)* strains, pre-treated with 50 ng/ml ATc for 4 days, at 1: 2 multiplicity of infection (macrophage: bacteria) for 4 h. Determination of intracellular growth by CFU counting suggests that despite equal infection on day 0 by both the control and *gyr(-)* strains, the latter shows significant (*P* < 0.01) attenuation on successive days, whereas the control Mtb maintains its viability in the resting macrophages at all time points (Figure 1D). In comparison to the control strain, viability of *gyr(-)* is reduced by 90% on day1, 86% on day2, and 72% on day3 of infection. These results thus suggest that gyrase depleted Mtb is unable to face the intracellular host conditions.

### Analysis of Cell Morphology of *gyr(-)*

**Figure 2:**
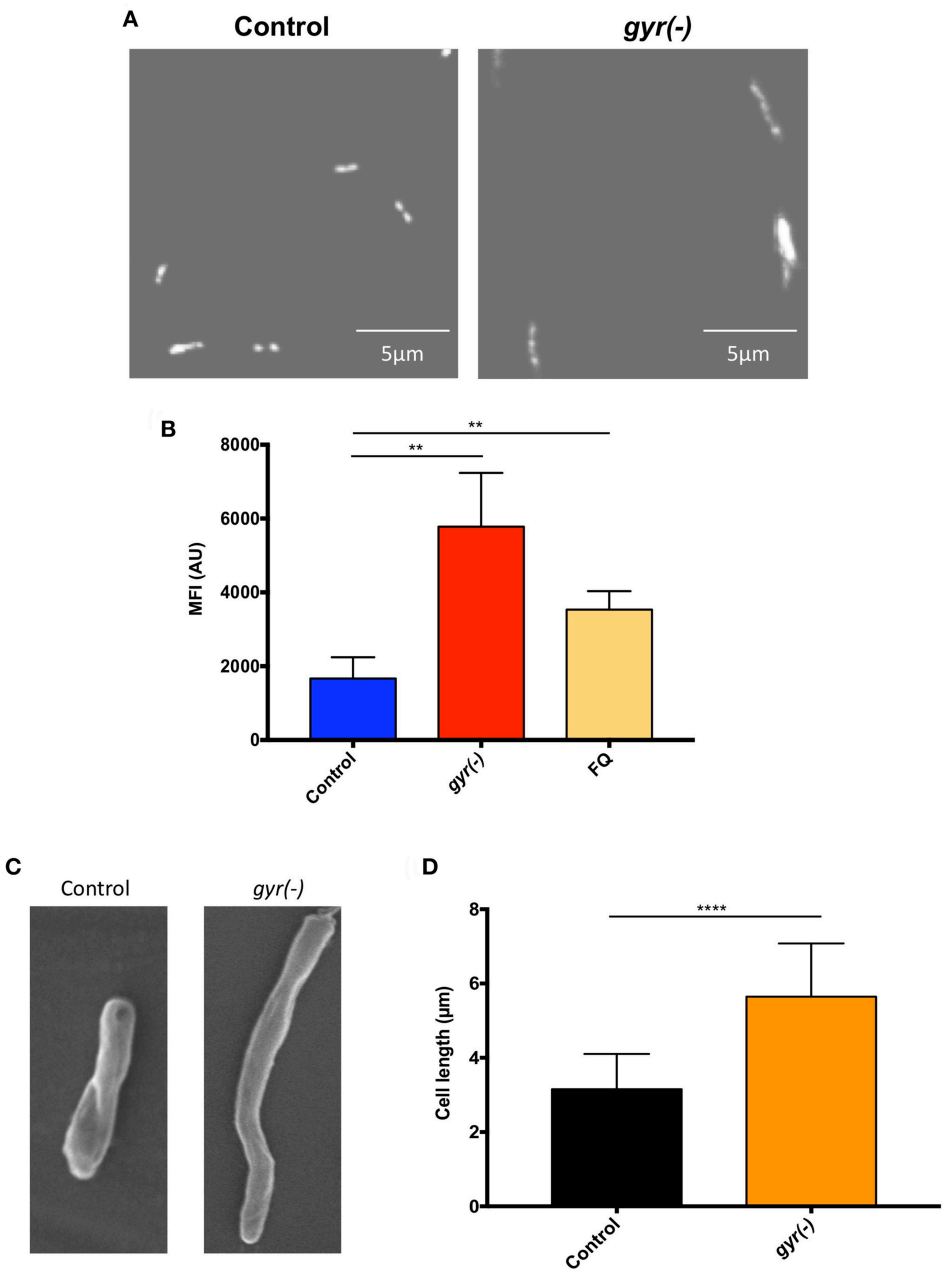
Depletion of DNA gyrase perturbs nucleoid morphology. **(A)** Effect of gyrase depletion on nucleoid organization. Morphology of 4′,6-diamidino-2-phenylindole (DAPI)-stained nucleoid of both control and *gyr(-)* was observed by confocal laser scanning microscopy using 100x oil objective under ultraviolet illumination. Each image is a representative of multiple fields from three different experiments. **(B)** Quantitative estimation of bacterial nucleoid decompaction. Status of DAPI-stained nucleoids of control and *gyr(-)* was analyzed by estimating median fluorescence intensity (MFI) of the dye using flow cytometry. Values were obtained from 10,000 events using FlowJo software (FlowJo LLC). FQ-treated Mtb was used as a reference control. Error bars represent SD from at least 3 replicates and statistical significance is determined by paired Student's *t*-test: ^**^P < 0.01. **(C)** Scanning electron microscopy (SEM) analysis of bacteria. Shown are the SEM images of control and *gyr(-)* after 4 days of treatment with 20 ng/ml ATc. Each image is a representative of multiple fields from three different experiments. **(D)** Effect of gyrase depletion on bacterial cell length. Cell length of control and *gyr(-)* was determined under light microscopy using 100x oil objective. Shown are the mean cell lengths of ~300 cells from three different experiments. Statistical significance is determined by paired Student's *t*-test: ^****^P < 0.0001.

In order to gain an insight into the effect of genetic suppression of DNA gyrase on Mtb growth, we further examined the nucleoid morphology of *gyr(-)* by confocal laser scanning microscopy using DNA-binding fluorescent dye, 4′,6-diamidino-2-phenylindole (DAPI), as reported earlier (Bhowmick et al., 2014). As depicted in Figure 2A, the *gyr(-)* shows a significantly dispersed nucleoid as against lobed pattern observed with the control Mtb (Figure 2A). Since defect in nucleoid morphology results in altered DAPI fluorescence intensity (Cho et al., 2004), the effect of gyrase depletion was also determined by fluorescence-activated cell sorting (FACS) analysis of DAPI-stained bacteria. A ~3.5-fold increase in the fluorescence intensity of DAPI from *gyr(-)* compared to control (*P* < 0.01) indicates perturbations of nucleoid architecture in *gyr(-)* (Figure 2B). Notably, a similar effect on DAPI fluorescence intensity was also observed with wild-type bacteria treated for 24 h with 5 μg/ml ofloxacin, a specific inhibitor of DNA gyrase (Figure 2B, FQ). Moreover, these effects are specific to DNA gyrase since we fail to observe any change in nucleoid morphology of Mtb depleted with unrelated essential gene *clpP2* (Figure S2).

Alteration in the nucleoid morphology is reminiscent of the aberrant replication status of a cell (Misra et al., 2018). Morphological analysis by scanning electron microscopy indicates a significant increase in bacterial cell length due to DNA gyrase depletion (Figure 2C). The mean cell length of control bacteria is 3.2 ± 0.05 μm, whereas the *gyr(-)* strain exhibits an 80% increase in cell length measuring to 5.7 ± 0.08 μm (*n* = 320; *P* < 0.0001; Figure 2D). As a positive control, we also determined the cell length of Mtb lacking the expression of a known cell division gene, *ftsZ* (Dziadek et al., 2003; Weiss, 2004). Similar to our observation with *gyr(-)*, bacteria depleted with *ftsZ* exhibits >100% increase in length compared to control (mean cell length = 6.9 ± 0.15 μm, *P* < 0.0001; Figure S3).

### Identification of DNA Gyrase Regulons by Whole Genome Transcriptional Profiling

There is an interplay between supercoiling state of genome and its transcriptional turnover (Willenbrock and Ussery, 2004; Ma and Wang, 2016). Since DNA gyrase is a type II topoisomerase which is involved in maintaining the topological state during major DNA transactions, it becomes prudent to study the aftermath of tempering with gyrase expression on the profile of mRNA transcripts. We therefore examined the global transcriptional response of Mtb to loss of DNA gyrase, achieved by genetic approach. Complementary DNAs (cDNAs) prepared from gyrase depleted or control cells, were labeled with alexa fluor dyes during the cDNA synthesis followed by hybridization with Mtb microarrays, as described in the materials and methods. Fold change in expression was calculated for each gene by comparing the spot intensities in both the control and *gyr(-)* after normalization with their respective total fluorescence intensities.

**Figure 3:**
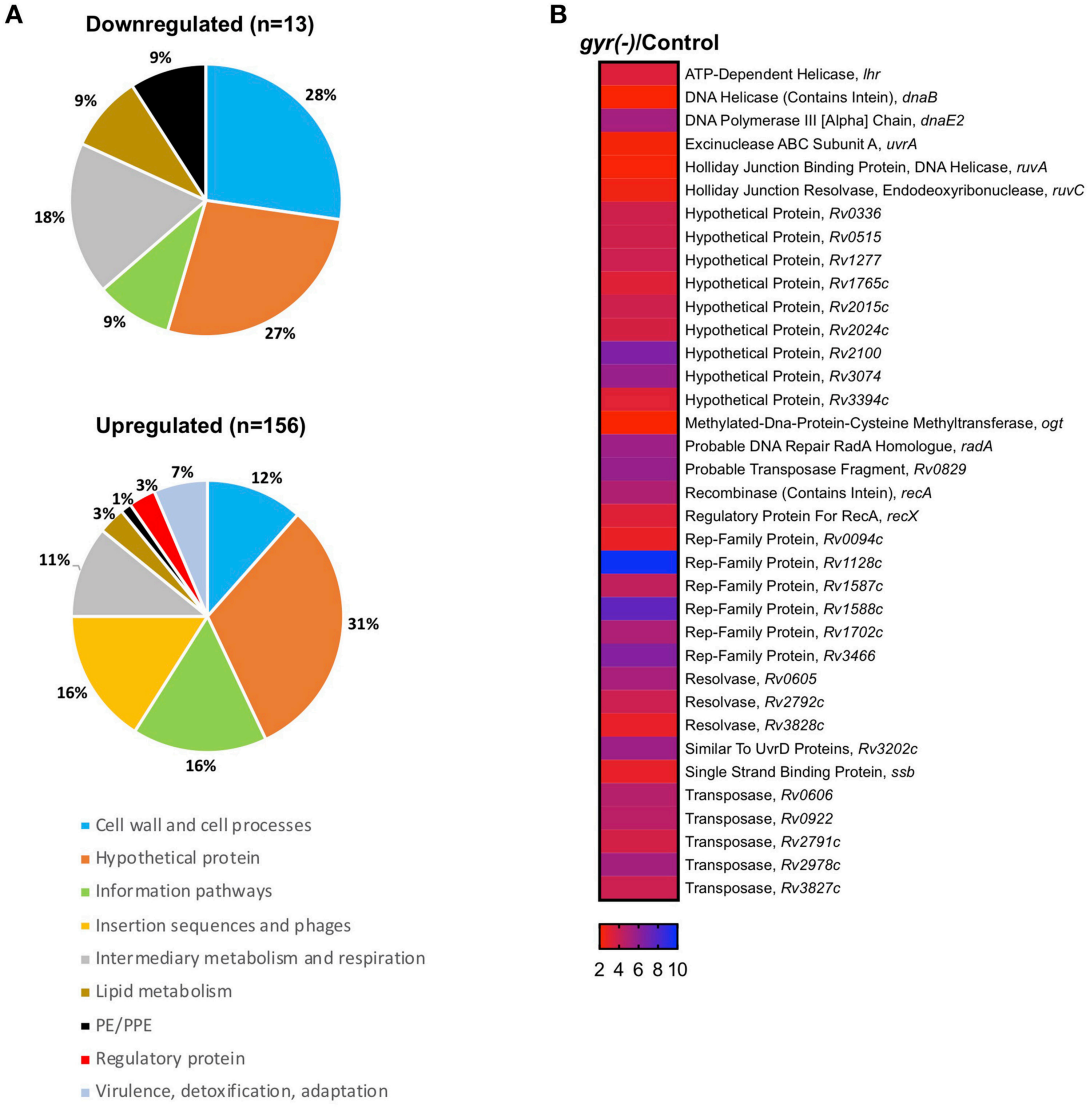
Silencing the DNA gyrase results in induction of DNA damage response in Mtb. **(A)** Microarray analysis of differentially expressed genes in *gyr(-)*. Pie charts show percentage distribution of differentially expressed genes in *gyr(-)* among various functional categories according to the classification by Mycobrowser database (https://mycobrowser.epfl.ch/genes/). Data represent two independent microarray experiments, each containing triplicate values for 3,924 genes. Shown are genes that are modulated by > 2-fold in *gyr(-)* compared to control Mtb (*P* < 0.05, as determined by paired Student's *t*-test and FDR of 5%). **(B)** Expression analysis of genes involved in bacterial DNA damage response in *gyr(-)*. Heat map shows a subset of genes from **(A)** known to control DNA damage response in Mtb (Boshoff et al., 2003).

Strikingly, 92% of the differentially expressed genes (*n* = 156) are significantly induced (>two-fold with *P* < 0.05 and false discovery rate (FDR) of 5%), whereas only a small fraction (13 genes) exhibit reduced expression (<0.5-fold with *P* < 0.05 and FDR of 5%) upon DNA gyrase depletion in Mtb (Supplementary Dataset 1 and Figure 3A). Functional classification of upregulated genes, as defined in the Mycobrowser database (https://mycobrowser.epfl.ch/), further reveals that genes corresponding to the information pathways (16%, *n* = 25), insertion sequences and phages (16%, *n* = 25), cell wall and cell processes (12%, *n* = 18), and intermediary metabolism and respiration (11%, *n* = 17) are primarily affected in *gyr(-)*. In addition, a significant proportion of uncharacterized genes (~31%) together with a small subset of genes involved in virulence, lipid biosynthesis and transcription are also upregulated in Mtb depleted with DNA gyrase (Figure 3A). Remarkably, 36 out of 59 genes involved in regulating bacterial SOS response under the conditions such as UV irradiation, treatment with hydrogen peroxide and a mutagenic agent mitomycin C (MMC) (Boshoff et al., 2003) are induced in Mtb upon DNA gyrase depletion (Supplementary Dataset 2 and Figure 3B). The list also includes a distinct set of transcripts viz., *Rv0336* (3.70-fold), *Rv0515* (3.71-fold), *Rv1702c* (5.02-fold), *Rv2100* (6.5-fold), *ruvC* (2.43-fold), *Rv2719c* (2.20-fold), *recA* (4.86-fold), *Rv3074* (5.66-fold), and *dnaE2* (5.27-fold) that are controlled by LexA repressor, a regulator of SOS response, in a RecA-dependent manner (Davis et al., 2002). The observed induction of the LexA regulons in *gyr(-)* by microarray hybridization was also verified by qRT-PCR using specific primers (Figure S4). Overall, the above results suggest that depletion of gyrase expression elicits SOS response in Mtb.

### Depletion of DNA gyrase in Mtb Induces Persisters

Induction of SOS response is correlated with the formation of persister cells in bacteria (Dörr et al., 2009). As we have observed significant induction of SOS-responsive genes in *gyr(-)*, we next determined the susceptibility of *gyr(-)* to the first-line anti-TB drugs. Broth cultures of control and DNA gyrase depleted bacteria with equal OD_600_ of 0.05 were treated with different concentrations of Rif, INH and Emb followed by bacterial enumeration using CFU plating (Supplementary Dataset 3).

**Figure 4:**
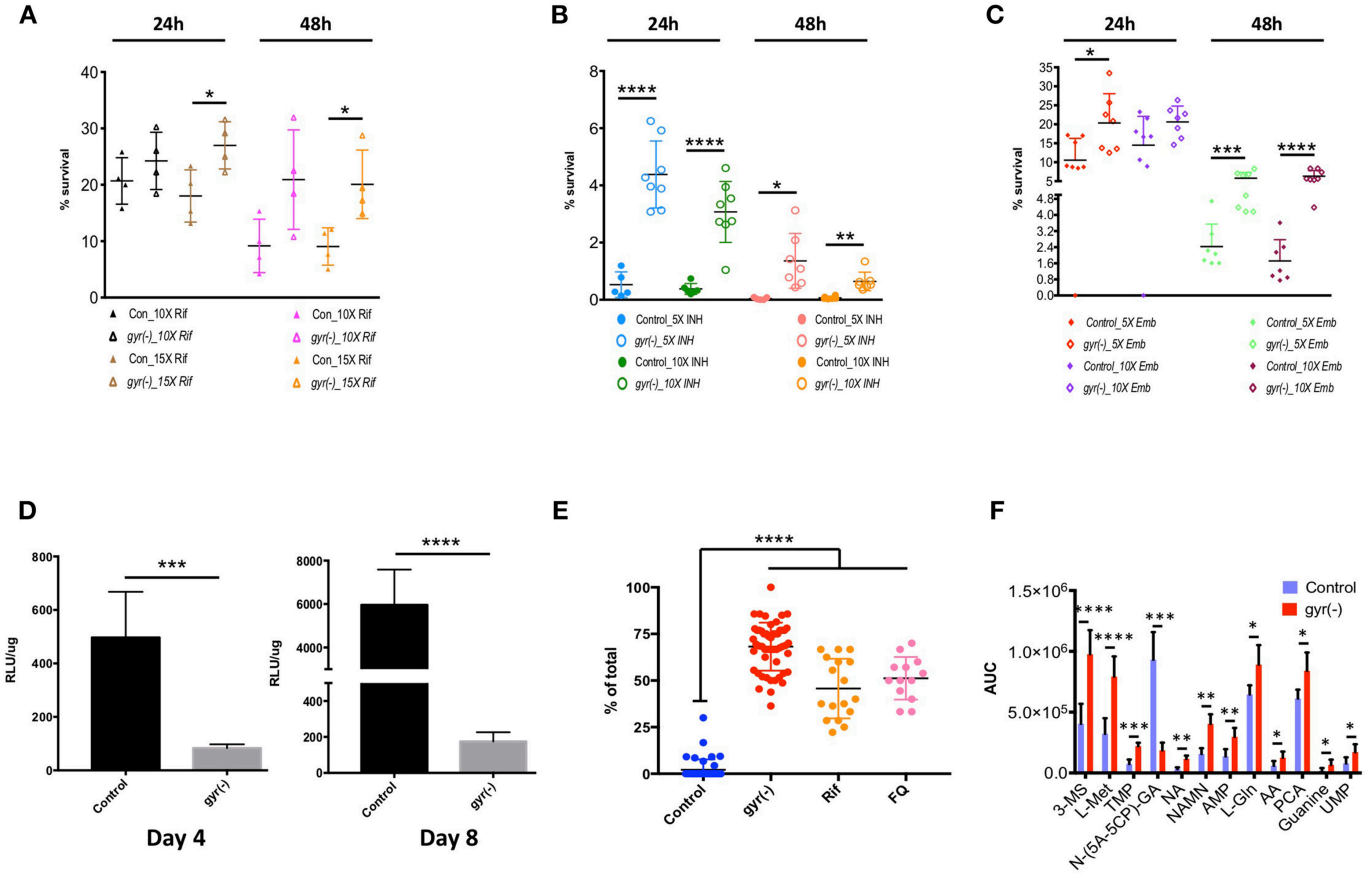
Depletion of DNA gyrase induces persister formation. Antibiotic susceptibility of gyrase knockdown strain to the first-line anti-TB drugs was determined by exposing bacterial cultures of control and *gyr(-)* at OD_600_ of 0.05 to different concentrations of drugs followed by CFU enumeration. Cell survival is expressed as percentage survival relative to drug-untreated cells. Reference minimum inhibitory concentration (MIC) of each drug against wild-type Mtb was obtained from the published literature. Shown are the dot-plots exhibiting the time- and dose-dependent survival kinetics of both the strains after treatment with: **(A)** Rif, **(B)** INH, and **(C)** Emb. Error bars represent the SD from at least four different measurements. Statistical significance is determined by paired Student's *t*-test: ^*^P < 0.05, ^**^P < 0.01, ^***^P < 0.001, and ^****^P < 0.0001. **(D)** Estimation of intracellular ATP levels. Intracellular ATP levels were measured in control and *gyr(-)* after 4- and 8 days of treatment with 20 ng/ml ATc by using BacTiter-Glo™ microbial cell viability assay (Promega). Final ATP levels were calculated after normalizing the intensity of luminescence (expressed as relative luminescence unit [RLU]) with total protein concentration in each sample. Error bars represent the SD from at least three different experiments. Statistical significance is determined by paired Student's *t*-test: ^***^P < 0.001 and ^****^P < 0.0001. **(E)** Estimation of lipid bodies. Lipid bodies (LB) were estimated in bacteria stained with Nile red by counting lipid-rich foci under confocal laser scanning microscopy using 100x oil objective. The dot plot shows percentage of total cells exhibiting >2 LBs in control, *gyr(-)*, Rif- and FQ-treated wild-type Mtb, respectively. Data were obtained by estimating LBs in at least 200 bacterial cells from multiple fields. Error bars represent the SD from three different measurements. Statistical significance is determined by paired Student's *t*-test: ^****^P < 0.0001. **(F)** Comparative analysis of metabolites in control and *gyr(-)* strains of Mtb. Quantitative estimation of metabolites in control and *gyr(-)* was performed by LC-MS/MS and average values from six different experiments are plotted in the bar graph. Shown are the metabolites that are modulated by >1.3-fold in *gyr(-)* compared to control Mtb. Statistical significance is determined by paired Student's *t*-test: ^*^P < 0.05, ^**^P < 0.01, ^***^P < 0.001, and ^****^P < 0.0001.

Our results reveal that Mtb lacking the expression of DNA gyrase exhibits 1.5–2.2-fold (*P* < 0.05) less killing compared to the control by Rif treatment with a 15-fold higher dose of drug than its minimum inhibitory concentration (MIC) for 24- and 48 h, respectively (Figure 4A). Similarly, the related tolerance pattern in *gyr(-)* is also seen toward other drugs such as INH and Emb (Figures 4B,C). While incubation with 5- and 10x MIC of INH results in >99.5% killing of control bacteria at both the time points, a significant proportion of *gyr(-)* cells exhibits tolerance under the same treatment conditions. As against control Mtb, the *gyr(-)* strain shows 8- (*P* < 0.0001) and 45-fold (*P* < 0.05) more survival after treatment with 5x MIC of INH for 24- and 48 h, respectively; the effect is similar even with higher drug concentration of 10xMIC where we find 8- (*P* < 0.0001) and 10-fold (*P* < 0.01) better survival of *gyr(-)* after 24- and 48 h of exposure, respectively (Figure 4B). Similar to these drugs, treatment with Emb also indicates significantly better tolerance of *gyr(-)* than control after 48 h of treatment with 5x (2.4-fold; *P* < 0.001) and 10x (3.7-fold; *P* < 0.0001) MIC of the drug (Figure 4C). Tolerance of DNA gyrase depleted Mtb to drugs was also confirmed by spotting the serially diluted bacterial cultures after drug treatment on 7H11-OADS agar (Figure S5A). Remarkably, level of tolerance is maintained in these bacteria even after successive passaging of the respective antibiotic-treated cultures (Figures S5B–E). In addition, these effects are specific to the knockdown of DNA gyrase, as suppression of another essential gene, *clpP2* does not alter bacterial susceptibility to antibiotics (Figure S6). In pursuit of determining whether drug tolerance is attributed in gyrase knockdown strain at the genetic level, we determined the MIC as well as killing effect of the first-line drugs, Rif, INH, and Emb using the passaged cultures of control and *gyr(-)*, but in the absence of ATc when no suppression of gyrase expression is observed. Our results show equal susceptibility of both the strains to these antibiotics (Figures S7A–E), thus suggesting that the apparent tolerance of DNA gyrase depleted Mtb to drugs is not genetically heritable.

The above results provide an indication of the presence of persisters in DNA gyrase depleted bacterial population that exhibit phenotypic drug tolerance. Since, energy state of bacteria plays an important role in maintaining persisters (Shan et al., 2017), we further determined the levels of ATP in Mtb following the DNA gyrase depletion. Total ATP was measured after 4 and 8 days of ATc treatment in empty vector containing control and *gyr(-)*. Intracellular ATP level was measured as described in the materials and methods, and the values of relative luminescence unit (RLU) were normalized with the total protein concentration in the respective samples. As shown in Figure 4D, total cellular ATP pool in *gyr(-)* is declined by six-fold (*P* < 0.0001) compared to control after 4 days of suppression, and this level is further reduced to 34-fold lower than control on day 8 of ATc treatment. Importantly, the control bacteria exhibit >10-fold increment in intracellular ATP level upon progression of *in vitro* growth from day 4 to day 8, whereas the same is changed by only two-fold in *gyr(-)* strain of Mtb (Figure 4D). Another hallmark of persisters in mycobacteria is the accumulation of lipid bodies (LBs). Recently it was shown that as high as 40-fold more drug concentration is required to clear lipid-rich mycobacteria (Hammond et al., 2015). Consistent with these findings, we also observe that wild-type bacteria treated with drugs such as Rif or FQ exhibit significant (*P* < 0.0001) elevation of LBs. Twelve hours of exposure to 10xMIC of Rif induces 22-fold increase in the number of LB+ bacteria (45.7 ± 3.9% LB+, *P* < 0.0001), whereas with FQ treatment under similar conditions, their levels are increased by 24-fold (51.2 ± 3.2% LB+, *P* < 0.0001), compared to drug-untreated bacteria (Figure 4E). These observations together indicate that formation of LBs is an inherent response of persister bacilli. Intrigued with these findings, we further evaluated the status of these lipid-rich structures in DNA gyrase depleted Mtb. Our results reveal that a significant proportion of *gyr(-)* bacteria (68.2 ± 1.8%, *P* < 0.0001) are loaded with LBs reminiscent of drug-tolerant persister population, in contrast to control strain where majority of the cells (97.9 ± 0.8%, *P* < 0.0001) are devoid of any such feature (Figure 4E).

### Effect of DNA Gyrase Depletion on Metabolic State of Mtb

In order to analyze the metabolic state of Mtb depleted with DNA gyrase expression, we estimated the levels of metabolites by LC-MS/MS, as described in the materials and methods. Metabolites in both the control and *gyr(-)* were quantified by using concentration-dependent standard curves for each metabolite as described earlier (Gupta et al., 2018). Those small molecules which exhibit minimal variation (*P* < 0.05) in six biological samples were examined for fold-change in their levels between the two strains (Supplementary Dataset 4). As presented in Figure 4F, we find 12 such metabolites that are differentially accumulated by >1.3-fold. Interestingly, DNA gyrase knockdown results in accumulation of most of these except N-(5-Amino-5-carboxypentyl)glutamic acid [N-(5A-5CP)-GA], which is an intermediate of lysine metabolic pathway, and exhibits ~five-fold reduction in *gyr(-)* compared to control. We observe overabundance of several nucleotides such as thymidine 5′-monophosphate (TMP, 3.0-fold), adenosine 5′-monophosphate (AMP, 2.18-fold), guanine (3.42-fold), and uridine monophosphate (UMP, 2.19-fold) in DNA gyrase depleted bacteria. Moreover, we also find significant increase in the levels of other metabolites in *gyr(-)* such as coenzymes nicotinic acid (NA, 3.61-fold) and nicotinic acid mononucleotide (NAMN, 2.58-fold) that are key regulators of energy metabolic pathways; and amino acids such as L-(-)-methionine (L-Met, 2.46-fold), L-glutamine (L-Gln, 1.38-fold), and L-pyroglutamic acid (PCA, 1.37-fold), respectively (Figure 4F). Overall, accumulation of these metabolites suggests that suppression of DNA gyrase results in perturbation of the central metabolic pathways in Mtb.

### Treatment With Suramin Augments Bactericidal Effect of Antibiotics Against *gyr(-)*

So far, our results have demonstrated that bacteria depleted with DNA gyrase exhibit altered nucleoid morphology and cessation of core metabolic pathways. Such metabolic perturbations eventually result in induction of the RecA-LexA dependent SOS response and phenotypic tolerance to anti-TB drugs in a subpopulation containing excess of lipid granules. These observations further prompted us to evaluate the effect of suppression of RecA-LexA mediated SOS response on formation of persisters in Mtb.

Few years ago, it was reported that suramin (also called germanin), which is in clinical use since the 1920s as an anti-trypanosomal (sleeping sickness) and anti-filiarial drug (Wang, 1995), inhibits ATPase and DNA strand exchange activities of *E. coli* RecA (Wigle and Singleton, 2007). Subsequently, the effect of suramin was also investigated against the RecA protein of Mtb, which showed that the drug not only adversely affects its activity by disassembling RecA–single-stranded DNA filaments, but it also blocks the elicitation of SOS response in FQ-treated *M. smegmatis* (Nautiyal et al., 2014).

**Figure 5:**
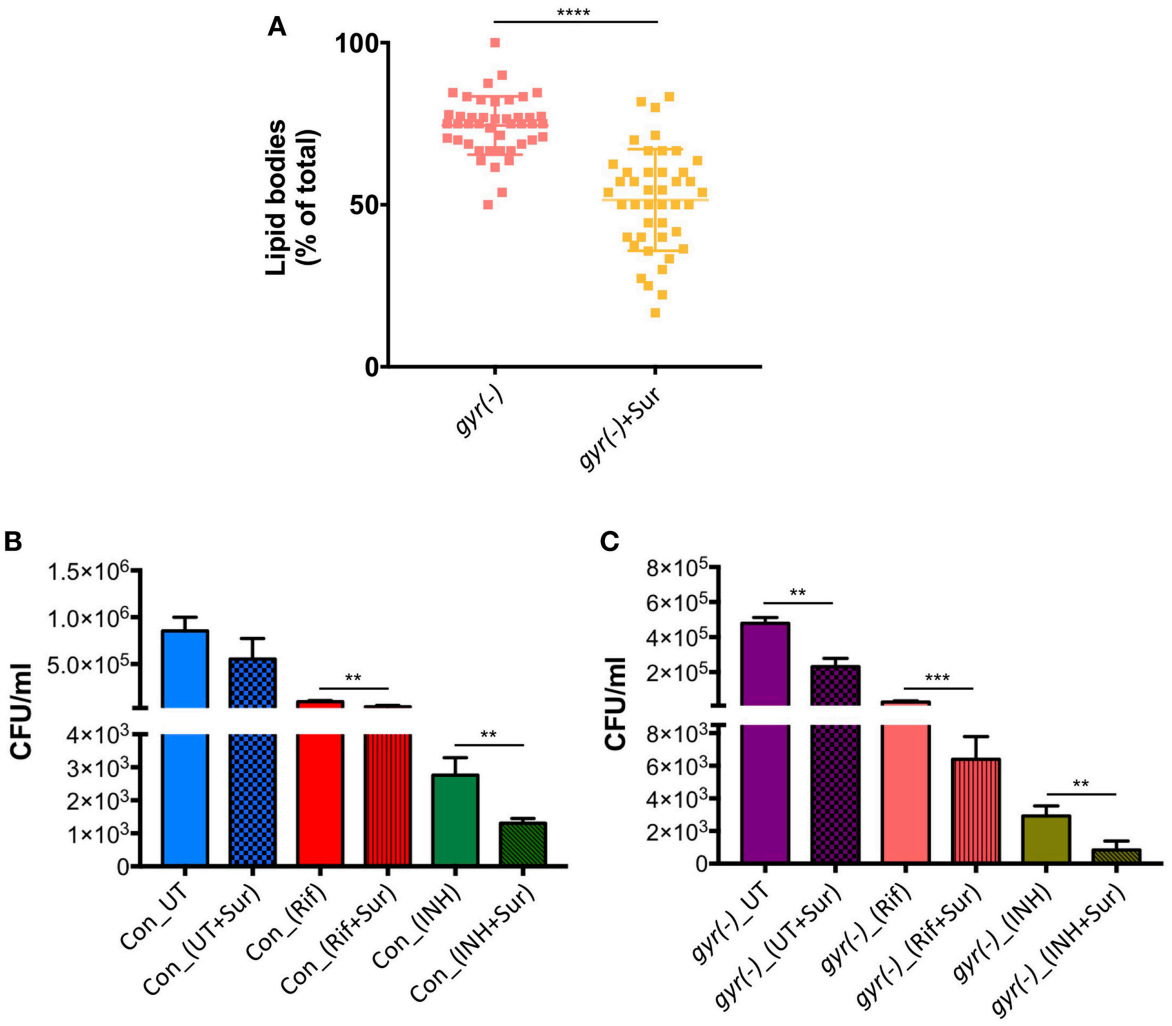
Inhibition of RecA potentiates the bactericidal effect of anti-TB drugs. **(A)** Effect of suramin on the induction of LBs in *gyr(-)*. DNA gyrase depletion was performed in the absence or the presence of 50 μM suramin for 4 days, followed by Nile red staining of cultures. Percentage accumulation of LB+ cells was estimated from at least 40 fields from a total of three independent experiments. Statistical significance is calculated by student's *t*-test: ^****^P < 0.0001. **(B)** Effect of suramin on the bactericidal effect of antibiotics against control Mtb. Survival of bacteria after 48 h of treatment with 10X MIC of Rif and INH in the absence or the presence of 50 μM suramin, respectively, was determined by CFU enumeration. Error bars represent the SD from four measurements. Statistical significance is calculated by student's *t*-test: ^**^P < 0.01. **(C)** Effect of suramin on the efficacy of drugs against *gyr(-)*. DNA gyrase depletion was performed in the absence or the presence of 50 μM suramin for 4 days, and its susceptibility against Rif and INH was determined by estimating CFU counts after 48 h of treatment with 10X MIC of drugs, with or without 50 μM suramin. Error bars represent the SD from four measurements. Statistical significance is calculated by student's *t*-test: ^**^P < 0.01, ^***^P < 0.001. CFU counts of untreated (UT) and suramin-treated cultures in the absence of drugs (UT+Sur) are included to estimate the augmented effect of suramin on bactericidal activity of anti-TB drugs against control **(B)** and *gyr(-)* **(C)**.

In order to examine if suramin treatment affects the overall frequency of persisters in Mtb, we analyzed its impact on accumulation of LBs in *gyr(-)*, and efficacy of Rif and INH against control and *gyr(-)* strains. Bacteria were treated for 4 days with a concentration of suramin (50 μM) which is lethal for RecA nucleoprotein filament assembly (Nautiyal et al., 2014), and LBs were counted as described above. Figure 5A shows a significant effect of suramin on accumulation of LBs in *gyr(-)*. While, 74.5 ± 1.4% of total mycobacterial cells exhibit the presence of LBs upon depletion of DNA gyrase, addition of 50 μM suramin in the bacterial culture of *gyr(-)* reduces their proportion to 51.0 ± 2.4% (*P* < 0.0001). Similarly, we find a marked improvement in killing (*P* < 0.01) of bacteria by Rif or INH when performed in the presence of suramin. Compared to Rif alone, treatment with Rif and suramin reduces the viability of control and *gyr(-)* by 2.3- and 4.6-fold, respectively, after 48 h of treatment. Likewise, bactericidal effect of INH is also found to be enhanced by 2.1- (against control) and 3.5-fold [against *gyr(-)*], when incubated in the presence of suramin for 48 h (Figures 5B,C).

## Discussion

Whilst FQ is clinically used as second-line anti-TB drug, its impact on the physiology of Mtb has never been evaluated. This is the first report to the best of our knowledge where we have successfully demonstrated the effects of DNA gyrase depletion including formation of drug-tolerant persisters in Mtb. In the wild-type cells replication of genomic DNA is dependent on supercoiling state at the site of replication fork. DNA gyrase facilitates the progression of replication fork by removing the positive supercoils without leaving nicks in the DNA molecule (Holmes and Cozzarelli, 2000; Aubry et al., 2006). However, due to depletion of DNA gyrase in the *gyr(-)*, its genome undergoes massive de-condensation possibly by accumulation of negative supercoils (Figure 2A). Error in DNA replication process subsequently halts cell division leading to significant reduction in bacterial viability (Figures 2C,D). Interestingly, after sharp decline in bacterial CFU counts during the initial 4 days period, growth of gyrase depleted Mtb strain is resumed at later time points, albeit with a slower rate, which suggests the elicitation of DNA repair mechanism in the proportion of viable bacteria (Figures 1C,D). Homeostasis of negative and positive supercoils is also a key factor in tuning the genomic transcription (Dorman, 1991; Dorman and Corcoran, 2009). Global transcriptional profiling reveals significant induction of genes controlling bacterial DNA damage response including those involved in DNA repair in *gyr(-)* on day 4 of depletion (Supplementary Datasets 1, 2, and Figure 3). These results together with decondensed nucleoid suggest that silencing the gyrase expression in Mtb is possibly causing damage of DNA molecules which subsequently results in significant loss of viability, although a parallel repair mechanisms has also initiated in the bacterial subpopulation that are viable and transcriptionally active. A thorough analysis of differentially regulated genes further reveals the elicitation of RecA-LexA mediated SOS response in these bacterial population of *gyr(-)* (Figure 3). Importantly, many of the persister regulons, as reported earlier (Keren et al., 2011), are also found activated in *gyr(-)*.

Arrested growth is presumed to be the prerequisite for the origin of persisters because these metabolically inferior population suffer limited damage from antibiotic treatment than their growing counterparts (Van den Bergh et al., 2017). Although, we find better survival of *gyr(-)* than control Mtb in the presence of antibiotics (Figures 4A–C), killing of a significant proportion of gyrase-depleted bacteria by drug treatment indicates the existence of heterogeneous population in *gyr(-)* comprising of drug-susceptible metabolically active cells as well as drug-tolerant metabolically inert persisters. It is intriguing how these two distinct population are intrinsically controlled in Mtb. Recently, it has been reported that survival of FQ-treated *E. coli* persisters is dependent on timing of DNA repair and resumption of DNA synthesis. Interestingly, it was observed that delayed onset of growth by carbon source starvation rescues almost 100% cells from killing by FQ treatment in a RecA-dependent manner (Mok and Brynildsen, 2018). These findings thus warrant future studies to gain a deeper insight into the mechanisms behind the formation of persisters in Mtb.

Persisters are characterized by their distinct respiratory status, differential accumulation of lipid granules and altered levels of metabolites (Garton et al., 2008; Allison et al., 2011; Orman and Brynildsen, 2013; Chuang et al., 2015; Lobritz et al., 2015; Sloan et al., 2015). Significant reduction in cellular ATP levels, abundance of lipid bodies and select metabolites such as nucleic acids, amino acids and coenzymes further support the presence of persisters in *gyr(-)* (Figures 4D–F). Remarkably, reduction in ATP despite accumulation of AMP further reiterates defect in cellular respiration of gyrase-depleted Mtb. Furthermore, accumulation of several nucleotides in *gyr(-)* could be a metabolic response of cells requiring translesion synthesis (Gon et al., 2011).

In *E. coli*, ciprofloxacin-induced persisters are formed in a SOS-dependent manner (Dörr et al., 2009). Since, RecA is the primary regulator of SOS response (Baharoglu and Mazel, 2014), we next determined the effect of RecA inhibition on the formation of persisters in Mtb. Since, simultaneous repression of *recA* and *gyr* in Mtb could not be achieved by CRISPRi, we used anti-filarial drug suramin, which targets RecA and blocks the elicitation of SOS response in bacteria (Wigle and Singleton, 2007; Nautiyal et al., 2014). Approximately 33% reduction in the formation of lipid bodies in suramin-treated *gyr(-)* and improved killing of Mtb by the anti-TB drugs in the presence of suramin provides an evidence for SOS-mediated induction of persisters in *gyr(-)* strain of Mtb (Figure 5). Remarkably, suramin alone does not exhibit antibacterial activity against control Mtb, whereas it inhibits growth of *gyr(-)* by ~two-fold. While the efficacy of Rif and INH is improved in the presence of suramin against both the control and *gyr(-)* strains, we find better effect under a condition when persisters are abundantly present (Figures 5B,C). Noteworthy to mention that we could not detect induction of RecA-LexA regulons by qRT-PCR in the control bacteria by Rif or INH treatment, which is possibly due to the abundance of drug-susceptible population, masking the effect produced by tiny proportion of drug-tolerant persisters under these conditions. Since suramin also exhibits inhibitory action against the purified DNA primase enzyme (DnaG) of *M. smegmatis* (Kuron et al., 2014), it is prudent to analyze the induction of persisters by knocking out the expression of *recA* gene in Mtb.

In summary, silencing the SOS-response by inhibiting RecA can be explored as an adjuvant strategy to potentiate the activity of the existing antimycobacterial agents and reduce the time course of TB treatment, which is one of the primary causes of accumulation of drug resistance in TB pathogens. Although both the avirulent and virulent strains of Mtb share a great deal of similarity, the absence of PhoPR regulons in Mtb H37Ra affect some of the key metabolic pathways such as lipid metabolism (Gonzalo Asensio et al., 2006), that could impact the bacterial response to gyrase depletion. Future studies are warranted to validate our findings in the virulent Mtb strain as well as systematically examine the effect of RecA inhibition on the efficacy of anti-TB drugs *in vitro* as well as in an animal infection model.

## Author Contributions

This study was designed by NA and EC. NA, EC, RS, and YK conducted the experiments. NA and EC wrote the manuscript and analyzed data with critical inputs from RS and YK.

## Accession Codes

Gene expression data have been deposited in GEO database under the accession codes GSE120139.

### Conflict of Interest Statement

The authors declare that the research was conducted in the absence of any commercial or financial relationships that could be construed as a potential conflict of interest.
