# Supplementary material for: Conditional Silencing by CRISPRi Reveals the Role of DNA Gyrase in Formation of Drug-Tolerant Persister Population in Mycobacterium tuberculosis
Source: Front Cell Infect Microbiol. 2019 Mar 26;9:70. doi: 10.3389/fcimb.2019.00070 (PMC6443821; doi:10.3389/fcimb.2019.00070)
Supplement: Supplementary file 2 [file Data_Sheet_1.PDF]

SUPPLEMENTARY INFORMATION

Conditional silencing by CRISPRi reveals the role of DNA gyrase in formation of drug-tolerant persister population in *Mycobacterium tuberculosis*

Eira Choudhary[1,2], Rishabh Sharma[1], Yashwant Kumar[1] and Nisheeth Agarwal[1]

Key words: *Mycobacterium tuberculosis*; Gyrase, DNA damage; SOS, Persisters

Running Title: Suppression of DNA gyrase induces persisters in *M. tuberculosis*

*To whom the correspondence should be addressed

Affiliation:
[1]Translational Health Science and Technology Institute,
NCR Biotech Science Cluster,
3rd Milestone, Faridabad–Gurgaon Expressway,
Faridabad- 121001 (Haryana)

[2]Symbiosis School of Biomedical Sciences,
Symbiosis International University,
Lavale,
Pune- 412115, (Maharashtra)

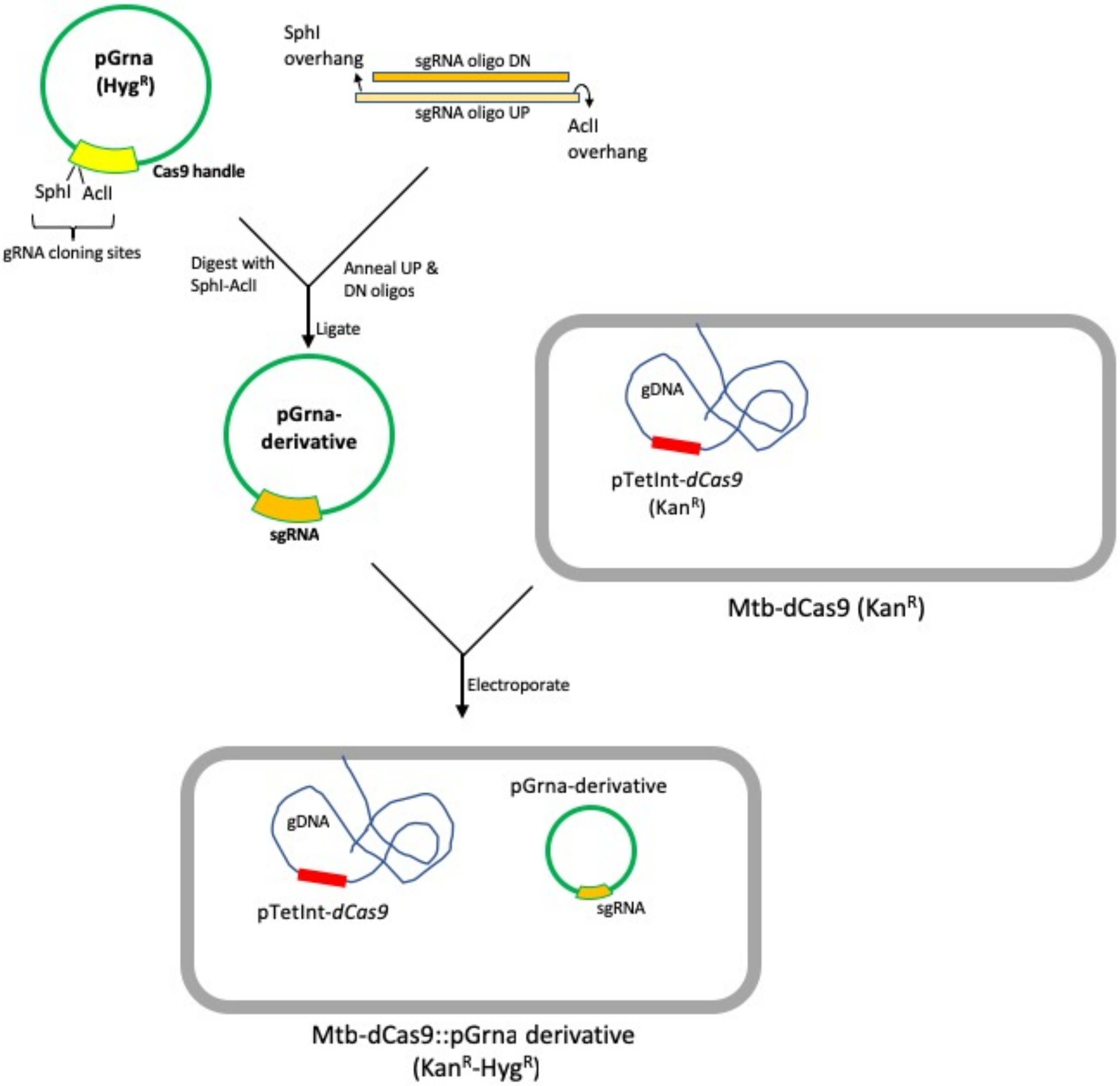

Figure S1: Schematic of construction of CRISPRi-based genetic knockdown strain in Mtb. Target-specific complementary oligonucleotides were designed such that after annealing they bear SphI and AclI overhangs. The annealed oligos were cloned at SphI-AclI sites, downstream to Tetracycline-inducible promoter and adjacent to Cas9 handle and Terminator in Hyg^R replicative plasmid pGrna. The recombinant derivative of pGrna was subsequently electroporated in Mtb harboring Kan^R integrative plasmid pTetInt-*dcas9* and colonies were selected on 7H11-Kan-Hyg. The resulting strain was treated with ATc to achieve desired level of suppression of target gene.

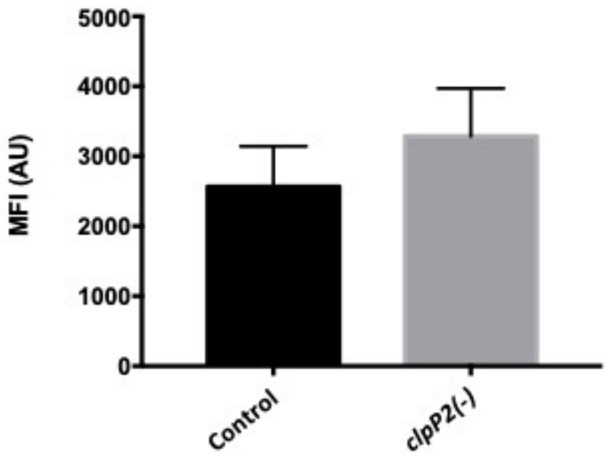

Figure S2: Effect of *clpP2* suppression in Mtb on nucleoid condensation. Median fluorescence intensity (MFI) of DAPI-stained bacteria was obtained by FACS which shows negligible difference (P= 0.07 by paired Student's t-test) in the nucleoid status between the control and the *clpP2(-)* strains. Values were obtained from 10000 events using FlowJo software (FlowJo LLC). Error bars represent SD from at least 3 replicates.

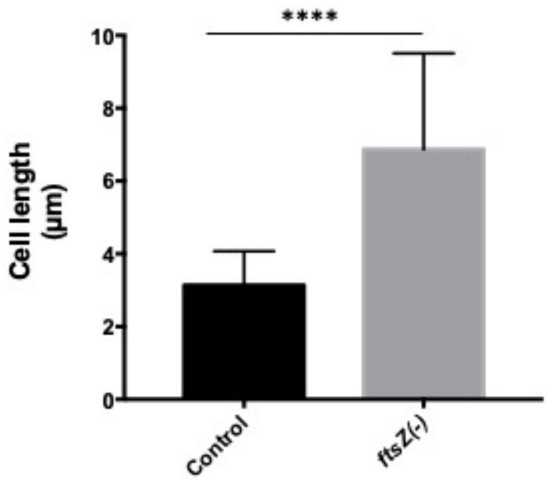

Figure S3: Genetic suppression of *ftsZ* adversely affects cell division of Mtb. Cell length of control and *ftsZ(-)* was determined under light microscopy using 100x oil objective. Shown are the mean cell lengths of ~300 cells from three different experiments. Statistical significance is determined by paired Student's t-test: **** $P < 0.0001$.

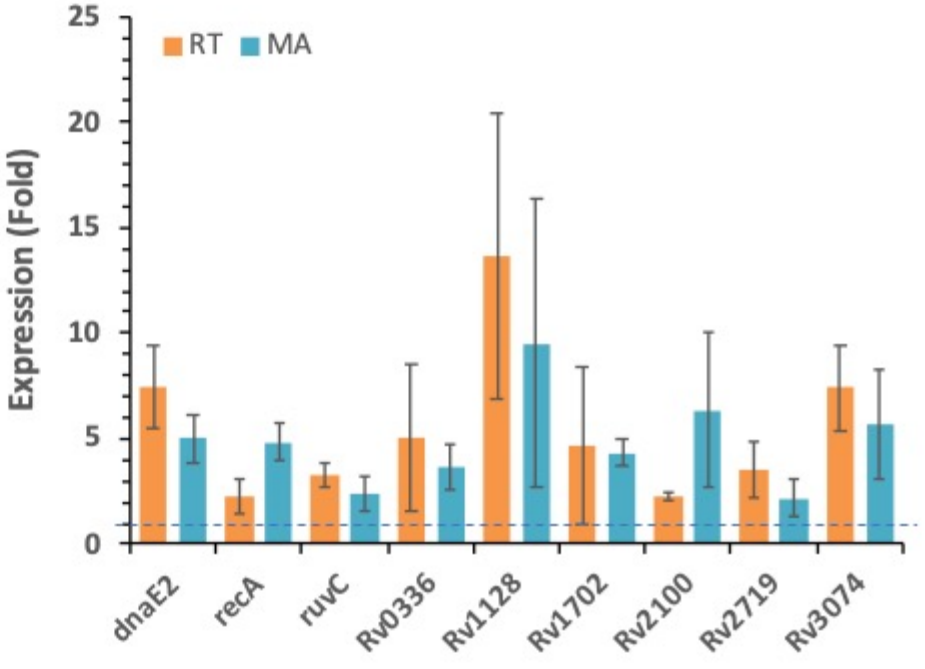

Figure S4: Verification of microarray data by quantitative real time reverse transcription PCR. Quantitative real time reverse transcription PCR (RT) was performed to analyze the change in expression status of LexA-regulons upon gyrase depletion, and compare with microarray (MA). Fold expression of a gene in ATc-treated bacteria relative to ATc untreated control was estimated after normalization with *sigA* levels under each condition. Error bars represent SD from 2 biological and 2 technical replicates. Statistical significance is determined by paired Student's t-test: $P \leq 0.05$. Broken line demarcates the base line.

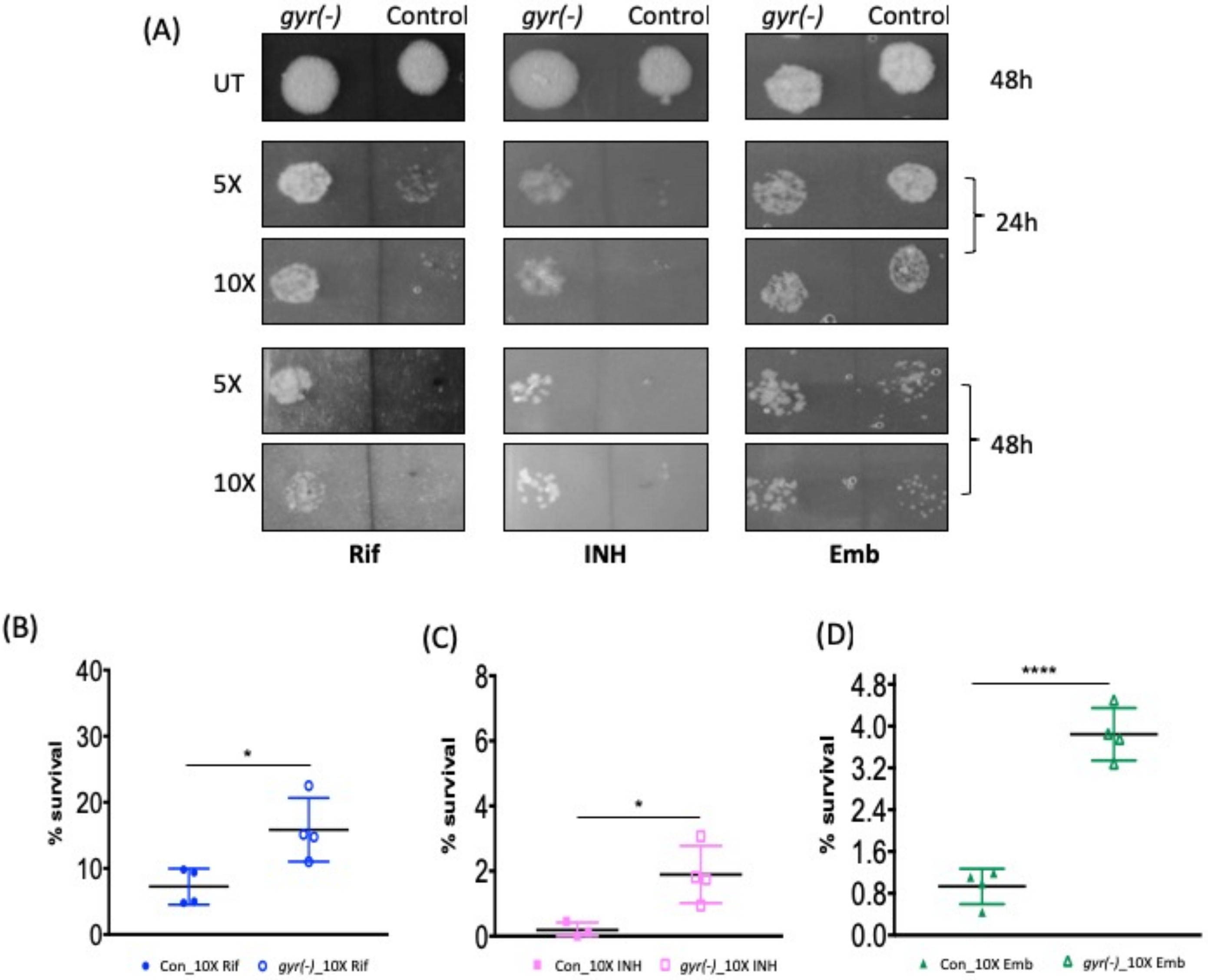

(E)

		Control	*gyr(-)*
**Rif (24hour)**	**0**	435200 ±154508	405760 ± 82279
	**10X**	28480 ±2186	63360 ± 19981
**INH (24hour)**	**0**	435200 ±154508	405760 ± 82279
	**10X**	640 ± 640	7360 ± 2587
**Emb (48hour)**	**0**	2026667 ± 616825	1242667 ± 378579
	**10X**	17600 ± 4710	47200 ± 13162

Figure S5: **Effect of DNA gyrase suppression on susceptibility of Mtb to antibiotics.** A) Analysis of *in vitro* antibiotic susceptibility of control and *gyr(-)* strains by spotting. Mtb cultures, untreated (UT) or treated with varying concentrations of drugs for different durations were spotted on 7H11 agar plates and growth was monitored after 4 weeks of incubation at 37ºC. (B-D) Analysis of *in vitro* antibiotic susceptibility of control and *gyr(-)* strains of Mtb after passaging of cultures. Shown are the effects of 10x MIC of Rif *(B)*, INH *(C)* after 24hours, and Emb *(D)* after 48hours of treatment. Antibiotic susceptibility was determined typically as described in Fig. 4. Survival is expressed as percentage survival in treated relative to drug-untreated cultures. (E) CFU analysis of bacteria after drug treatment of passaged cultures. Statistical significance is determined by Student's t-test: * $P<0.05$, **** $P<0.0001$. Image is representative of 3 experiments in (A).Data represent average of values from at least 3 experiments in (B-E).

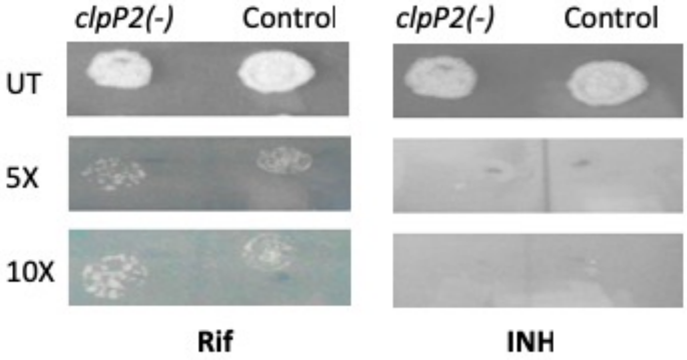

Figure S6: **Effect of *clpP2* suppression on susceptibility of Mtb to antibiotics.** Analysis of *in vitro* antibiotic susceptibility of control and *clpP2(-)* strains by spotting. Mtb cultures, untreated (UT) or treated with varying concentrations of Rif and INH for 24hours were spotted on 7H11 agar plates and growth was monitored after 4 weeks of incubation at 37ºC. Image is representative of 3 experiments.

(A)

	MIC $_{99}$ (µg/ml)	
	Control	*gyr(-)*
Rif	0.062	0.062
INH	0.125	0.125
Emb	8	8

(B) (C) (D)

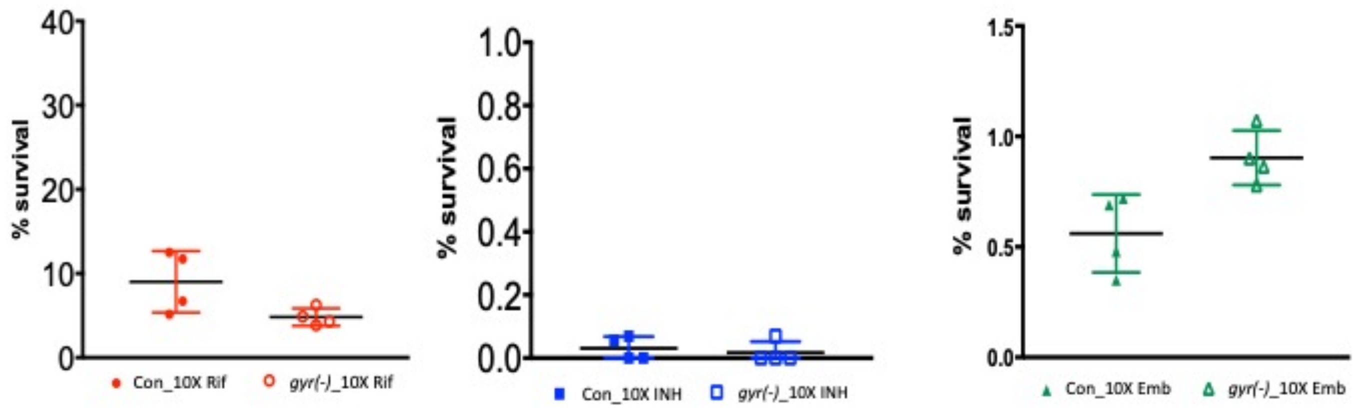

(E)

		Control	gyr(-)
Rif (24hours)	0	524800 ± 110723	939520 ± 338424
	10X	47520 ± 22527	44800 ± 15289
INH (24hours)	0	524800 ± 110723	939520 ± 338424
	10X	320 ± 0	480 ± 0
Emb (48hours)	0	4576000 ± 1862202	3429333 ± 877416
	10X	23600 ± 6581	30800 ± 7770

Figure S7: **Susceptibility of ATc-untreated cultures to antibiotics.** (A) MIC_{99} determination of antibiotics against ATc-untreated control and *gyr(-)* strains from the passaged cultures. MIC was determined by microtiter broth dilution, as mentioned in materials and methods. (B-D) *In vitro* antibiotic susceptibility of ATc-untreated cultures of control and *gyr(-)*. Shown are the effects of 10x MIC of Rif *(B)*, INH *(C)* after 24hours, and Emb *(D)* after 48hours of treatment. Antibiotic susceptibility was determined typically as described in Fig. 4. Survival is expressed as percentage survival in treated relative to drug-untreated cultures. (E) CFU analysis of ATc-untreated cultures of control and *gyr(-)* after drug treatment. Statistical analysis by Student's t-test shows no difference in survival of Rif and INH treated bacteria. Data represent average of values from at least 3 experiments in (A-E).

Table S1: List of oligonucleotides.

S. No.	Primer	Sequence (5'-3')	Reference
1.	Pr1	GTGCATGCGGTTGAGTCGCCG	This study (for cloning of *gyrB* specific sgRNAs)
2.	Pr2	CGCGGCGACTCAACCGCATGCACCATG	
3.	Pr3	GGACTCCTTGACCCCGAAG	This study (for cloning of *clpP2* specific sgRNAs)
4.	Pr4	CGCTTCGGGGTCAAGGAGTCCCATG	
5.	Pr5	CCACGACCTTGATGACGGC	This study (for cloning of *ftsZ* specific sgRNAs)
6.	Pr6	CGGCCGTCATCAAGGTCGTGGCATG	
7.	Pr7	ACGCCGGGTATTCGGAGTCG	This study (for qRT-PCR analysis of *gyrB*)
8.	Pr8	CTGCGGTTCGCTGACCTTCACC	
9.	Pr9	ACTGATCGTCGGATCCCAGG	This study (for qRT-PCR analysis of *gyrA*)
10.	Pr10	CAATGTTGGAAATGCCGGCC	
11.	Pr11	AAAAGGGTAAGCCGCGCTACG	This study (for qRT-PCR analysis of *dnaE2*)
12.	Pr12	ATGATGGGTCAGCTCGATGC	
13.	Pr13	GTTACGGCAAAGGTTCGGTGATGC	This study (for qRT-PCR analysis of *recA*)
14.	Pr14	ATGAACGCCGCAACACCACC	
15.	Pr15	GGCTGTCGCTTATCGAGAGTGG	This study (for qRT-PCR analysis of *ruvC*)
16.	Pr16	GCTGAGAGAACACCCGTTCG	
17.	Pr17	TGATGAGCGGTTTGAATGCC	This study (for qRT-PCR analysis of *Rv0336*)
18.	Pr18	TGTCCATCACCCACTCCTCG	
19.	Pr19	GTGTTCCACTCGGGAGGAGATCACG	This study (for qRT-PCR analysis of *Rv1128*)
20.	Pr20	TCCTCGGTGGATTGTTCGCC	
21.	Pr21	ATGTATTCGAGTAGCCGGGAGG	This study (for qRT-PCR analysis of *Rv1702*)
22.	Pr22	CGAGTTTGTTGATGAAGGGG	
23.	Pr23	TCGGTGGCCGCTACTAGTATCG	This study (for qRT-PCR analysis of *Rv2100*)
24.	Pr24	AGAAGTCACACGCCCAGTCG	
25.	Pr25	ATGTCGAGGACTGGGCATGG	This study (for qRT-PCR analysis of *Rv2719*)
26.	Pr26	ACGTCATACAGGGACTCCCC	
27.	Pr26	CGAGACATTGACCGCGATCG	This study (for qRT-PCR analysis of *Rv3074*)
28.	Pr27	TGAATCTCGTCGAGCCAGGG	
